# Editorial Comments: Is multiple sclerosis a neurodegenerative or inflammatory disease?

**DOI:** 10.1007/s00702-013-1044-7

**Published:** 2013-09-06

**Authors:** Amos D. Korczyn

**Affiliations:** Tel-Aviv University Medical School, Ramat Aviv, 69978 Israel


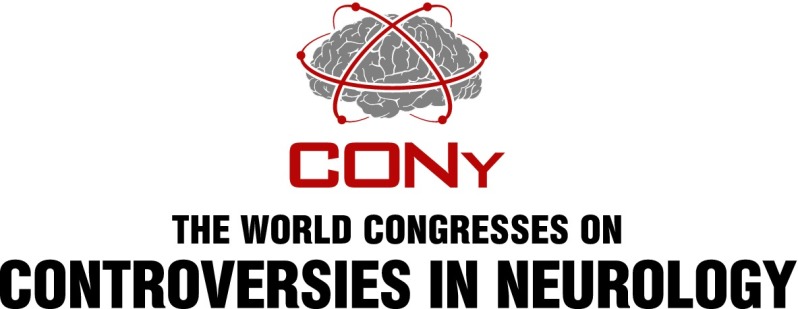


The etiology of multiple sclerosis (MS) and its pathogenesis are unclear in spite of being the topic of much research for many years. Neuropathological examinations provide undisputable evidence of inflammatory processes in acute demyelinating lesions in MS. This by itself is not surprising, since even if these inflammatory processes are secondary to the initial lesion, they may still have unfavorable consequences. In fact, the beneficial effects of high-dose steroid administration in speeding up the recovery from acute MS relapses seem to support this view.

The assumption that inflammation has a central role in the disease, rather than an epiphenomenon, has persisted for decades. This assumption that inflammation processes underlie the neurodegenerative process in MS was the basis for chronic therapy with steroids, which has been popular in the past. However, a more critical examination showed this to be an unrewarding treatment.

Inflammation is a term used to describe many processes, sometime complementing and at other time opposing each other, some beneficial and others not. Therefore, a systematic study regarding the role of inflammation in MS should be made in a more focused way. The positive effects of β-interferons (which are anti-inflammatory) support a damaging role of inflammation in acute relapses. However, by the same token, their inefficacy during the secondary progenies phase argues against an inflammatory role in the chronic stage.

The neurodegenerative component of MS may or may not be a consequence of inflammation. Neither corticosteroids nor specific anti-inflammatory agents like interferons are effective in the chronic progressive stage of MS. Whether other focused anti-inflammatory process modulation could be beneficial remains to be seen. However, the development of such drugs depends on a more refined definition of the inflammatory process components and discovery of specific targeted interventions. For the time being, the jury is still out: Is MS primarily an inflammatory disorder, where the neurodegeneration is a consequence, or is it primarily a neurodegenerative disorder, in which inflammatory responses are secondary, occurring at the beginning but later die out.

